# Reply to “comment on Post-vaccination incidence and side effects of COVID-19 in a cohort of Brazilian healthcare professionals: an internet-based survey”

**DOI:** 10.31744/einstein_journal/2023CE0572

**Published:** 2023-10-26

**Authors:** Matheus Ballestero, Renato Lucas Passos de Souza, Thiago Mamoru Sakae, Luiz Guilherme Villares da Costa, Luciano Furlanetti, Ricardo Santos de Oliveira

**Affiliations:** 1 Universidade Federal de São Carlos São Carlos SP Brazil Universidade Federal de São Carlos , São Carlos , SP , Brazil .; 2 Faculdade de Medicina de Ribeirão Preto Universidade de São Paulo Ribeirão Preto SP Brazil Faculdade de Medicina de Ribeirão Preto , Universidade de São Paulo , Ribeirão Preto , SP , Brazil .; 3 Universidade Federal de Santa Catarina Araranguá SC Brazil Universidade Federal de Santa Catarina , Araranguá , SC , Brazil .; 4 Hospital Israelita Albert Einstein São Paulo SP Brazil Hospital Israelita Albert Einstein , São Paulo , SP , Brazil .; 5 King’s College Hospital Charity London UK King’s College Hospital Charity , London , UK .

Dear Editor,

We appreciate the opportunity to respond to the comments raised in the letter to the editor regarding our article “Post-vaccination incidence and side effects of COVID-19 in a cohort of Brazilian healthcare professionals: an internet-based survey.” ^( 1 )^ We thank the authors for their interest in our study and for raising important points.

We agree with the authors that it is challenging to exclude symptoms resulting from other causes, rather than from the COVID-19 vaccine. Our study has acknowledged this limitation because it is difficult to distinguish between preexisting comorbidities and side effects of the vaccine. This is also consistent with findings from other studies that have highlighted the challenges in attributing symptoms to the adverse effects of the vaccines. ^( 2 , 3 )^

Furthermore, we agree with the authors that it is essential to continue monitoring adverse effects following COVID-19 vaccination. As vaccination programs continue to roll out globally, monitoring and reporting vaccine-related adverse effects are crucial to ensuring public trust in their safety and efficacy.

Despite these limitations, our study found a higher incidence of minor adverse effects, such as fever and headache, in individuals with a history of previous SARS-CoV-2 infection following COVID-19 vaccination. This finding is consistent with other studies that have reported an increase in adverse effects following the second dose of the vaccine, particularly in individuals with a history of previous infection. ^( 4 , 5 )^

We hope that our findings, as well as those of other studies reporting the adverse effects following COVID-19 vaccination, will encourage individuals to continue getting vaccinated against COVID-19 because it remains one of the most effective ways to mitigate the spread of the virus and to protect individuals and communities against severe illness and death.

Once again, we thank the authors for their valuable contribution and appreciate the opportunity to respond to their concerns.

Sincerely,
